# Friction-Stir Welding of a Wrought Al-Si-Mg Alloy in As-Fabricated and Heat-Treatment States

**DOI:** 10.3390/ma13040861

**Published:** 2020-02-14

**Authors:** Chunxia Wang, Hongbo Cui, Xin Tang, Kezhun He

**Affiliations:** 1 Key Laboratory of New Processing Technology for Nonferrous Metals & Materials, Ministry of Education, Guilin University of Technology, Guilin 541004, China; cuihb@glut.edu.cn (H.C.); xtang@glut.edu.cn (X.T.); 2 College of Materials Science and Engineering, Guilin University of Technology, Guilin 541004, China; 3 Guangxi Engineering and Technology Center for Utilization of Industrial Waste Residue in Building Materials, Guilin University of Technology, Guilin 541004, China; 4 Alnan Aluminium Co., Ltd., Nanning 530031, China

**Keywords:** Al-Si alloy, friction stir welding, heat-treatment, microstructure, mechanical properties

## Abstract

A wrought Al-11.3Si-0.6Mg alloy under hot extrusion (T1), solution treatment (T4), and solution treatment + artificial aging (T6) states were friction stir welded at welding speed of 100 mm/min and rotation rate of 800 rpm. The effect of prior heat-treatment on the microstructure and mechanical properties of the welds were investigated. The results show that the microstructures of the nugget zones have little dependence on the initial states of the base material. In the nugget zones, complete recrystallized structures with equaxied grains in the Al matrix were formed under all conditions. The Si particles in the nugget zones are almost unchanged compared with those of their base materials (BMs) in the three states. In contrast, the joint efficiency of the obtained welds was very sensitive to the initial material condition. The joint efficiency under the T1 state is more than 90% due to the fact that the microstructure is almost unchanged, except for the slight coarsening of the Al matrix grains and some of the Mg_2_Si phases during the friction stir welding process. However, the joint efficiency in the T4 and T6 conditions is only 77.22% and 62.03%, respectively. The relatively low weld strength in the T4 and T6 conditions is due to the elimination of the solid solution strengthening and age hardening effects during friction stir welding. The hardness distributions along the cross section of joints are all W-shaped under T1, T4, and T6 conditions.

## 1. Introduction

As a solid-state process, friction stir welding (FSW) has the advantages of great heat input, is free of filler metal and shielding gas, and avoids melting compared with the fusion welding processes and can produce high-quality joints in aluminum alloys [1,2,3,4]. Many researches have shown that FSW can produce fine microstructure, has an absence of welding defects, has low residual stresses, and has better dimensional stability in aluminum alloy joints [1,3,4]. In addition, better mechanical properties of FSW joints can also be obtained [5,6,7].

Al-Si alloys are an attractive engineering material because of their high electrical conductivity, low coefficient of thermal expansion, and light weight [8]. However, it is widely accepted that the cast Al-Si alloys, especially eutectic and hypereutectic Al-Si alloys, are brittle and hard to deform due to the coarse eutectic Si phases or primary Si particles, which are non-deformable and brittle. Recent studies found that Si phase refinement and massive Al grains can be achieved in Al-Si alloys through conventional plastic deformation (rolling, extrusion, and forging, etc.), which improves their plasticity, toughness, and strength and means that Al-Si alloys have the potential to be widely used as structural materials [9,10]. It has been reported that wrought Al-Si alloys can be welded using traditional fusion welding techniques. The microstructure of the welded zones is mainly a conventional solidification structure, containing fully developed Al dendrites and coarse inter-dendritic Al-Si eutectic structures after tungsten inert gas arc welding (TIG) [11]. Therefore, if traditional fusion welding techniques are used on wrought Al-Si alloys, the advantages of plastic deformation on cast Al-Si alloy would disappear and welding flaws, such as cracks, slag inclusions, and porosity, are easily formed [11]. In this case, FSW is a promising joining technique for wrought Al-Si alloys.

Friction heating and deformation are generated during the FSW process [1]. The plasticized soft materials are transferred from front to back through the rotating pin and shoulder, which leads to significant variations in microstructure, such as grain size, texture development, and precipitate distribution [12,13]. The welding strength is related to the microstructure changes during the FSW process. In non-heat treatable aluminum alloys, full-strength FSW joints can be obtained in initial annealed state [14,15]. The FSW joint strength is relatively low because of the recrystallization occurring for initial work hardened state alloys [16]. In heat-treatable aluminum alloys, the joint strength is dominated by the significant microstructural variations of the FSW joints in the size, density and distribution of the second phase precipitates [9,17,18,19]. In initial heat-treatable aluminum alloys, the dissolution or coarsening of reinforcement precipitates is due to the heat generation and lead to reduced weld strength during FSW [20]. Therefore, the material softening is closely related to the states of the base materials under FSW. However, research on the effect of the heat treatment states of the base materials on the microstructures and mechanical properties of the FSW joints of heat-treatable aluminum alloys is very rare.

Therefore, the purpose of this study is to evaluate the weldability of Al-Si alloys under different initial heat treatment states and to identify the effect of initial heat-treatment states on the mechanical properties and microstructure of the welded joints of an Al-Si-Mg alloy by FSW.

## 2. Experimental Procedure

An Al-Si-Mg alloy ingot was prepared with commercially pure aluminum (Baikuang, Baise, China) and silicon (SichuanLande, Chengdu, China) without chemical modification by direct-chill (DC) casting at a pouring temperature of 760 °C and a cast velocity of 200 mm/min. The chemical composition (wt.%) of the Al-Si-Mg alloy was: 11.3 Si, 0.6 Mg, 0.14 Fe, and Al balanced. The ingot was then extruded into plates, 4 mm in thickness, at 480 °C after pre-heat-treatment at 480 °C for 4 h.

The specimens, with a width of 50 mm and a length of 300 mm, were cut from the extrusion profiles for FSW. The extrusion specimens were marked as T1 state. Before the FSW process, T4 (solution treatment) and T6 (solution treatment + artificial aging) heat-treatment were carried out on a portion of the extrusion samples. The solution treatment was conducted at 520 °C for 1 h in a Muffle furnace (Michem, Beijing, China), and the samples were then quenched in room temperature water. Artificial aging was carried out at 180 °C for 3 h in a Muffle furnace. The plates with the same treatment states were butt-welded along the extrusion direction at a welding speed of 100 mm/min and a rotation rate of 800 rpm using an FSW machine. The welding tool was made of steel with an 18 mm diameter shoulder and a threaded and tapered triangle pin, 8 mm diameter at the root, 6 mm diameter at the head, and 3.5 mm in length. All FSW processes adopted a tilting angle of 2.5°, and the pressing depth of the shoulder was controlled at about 0.2 mm.

The FSW samples for macrostructure and microstructure observation were cross-sectioned perpendicular to the welding direction. The macrostructure and microstructure of the base and welded materials were analyzed by a Lecia S8 APO stereoscopy (Leica, Weztlar, Germany), a Lecia DMI8A optical microscope (OM) (Leica, Weztlar, Germany), a Zeiss mini 300 scanning electron microscope (SEM) (Zeiss, Orb Conhen, Germany) equipped with C-Nano electron backscattering diffraction (EBSD), an energy dispersive spectrometer (EDS), and a FEI Tecnain G2 F30 Transmission Electron Microscope (TEM) (FEI, Hillsboro, OR, USA) equipped with an EDS. The samples for the macrostructure and optical microstructure observations were polished and etched with Keller’s reagent. The average size of the Al matrix grains and Si phases was analyzed by the LAMOS Expert software on the Lecia DMI8A optical microscope (Leica, Weztlar, Germany). Samples for SEM and EBSD were prepared in the standard manner. Thin foils for TEM were prepared using double-jet electro polishing with an electrolyte of 30% nitric acid and 70% methanol operated at −20 °C and 15 V.

The Vickers hardness profiles of the joints were measured on the cross section perpendicular to the welding direction along the mid-thickness of the welds using a Leco AMH43 microhardness tester (Leco, St. Joseph, MO, USA) under a load of 100 g for 15 s with an interval of 0.5 mm. Transverse tensile samples with a gage length of 25 mm and a gage width of 6.25 mm were prepared in the direction perpendicular to the welding. Room temperature tensile tests were conducted at a loading speed of 1 mm/min on a SHIMADZU AG2-01 testing machine (Shimadzu, Kyoto, Japan) and three samples were tested for the various conditions. The fracture surfaces were examined on a Hitachi S-4800 SEM (Hitachi, Tokyo, Japan).

## 3. Results and Discussion

### 3.1. Microstructure of the Base Materials

**Figure 1:**
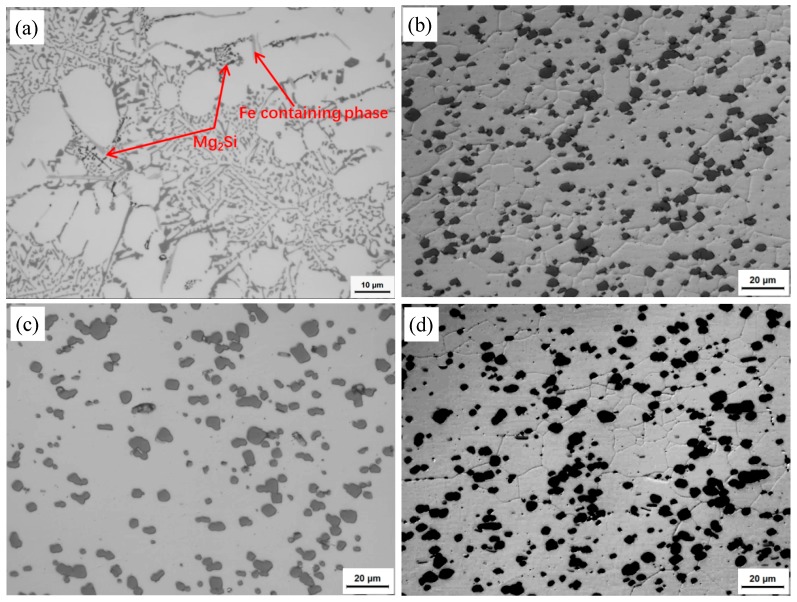
Optical micrographs under different states: (**a**) as-cast; (**b**) T1; (**c**) T4; (**d**) T6.

The optical micrographs of the as-cast and base materials (BM) under different states are shown in Figure 1. It can be seen that the microstructure of the DC cast alloy consists of fully developed α-Al dendrites, (α-Al + Si) eutectics and a small amount of black Mg_2_Si phases and gray Fe-containing phases [9,10]. The eutectic Si phases present as coral-like. It is obvious that the coral-like eutectic Si phases have been changed into fine Si particles with an average size of 3.01 μm, and the Si particles have been redistributed uniformly in the Al matrix with equiaxed grains after hot extrusion (Figure 1a,b). The distribution of the Al matrix equiaxed grains is inhomogeneous. In the concentrated silicon particle areas, the average size of the equiaxed grains is 7.47 μm. After T4 and T6 heat-treatment, it can be seen that the Si particles are more rounded and coarsened. The size of the Si particles is more uniform. The average size of the Al matrix grains and Si particles after T6 heat-treatment have been increased slightly to 7.87 μm and 3.42 μm, respectively, and the Al matrix grains are inhomogeneous under T6 heat-treatment. It should be noted that the size of the Si particles has not changed during the artificial aging treatment.

**Figure 2:**
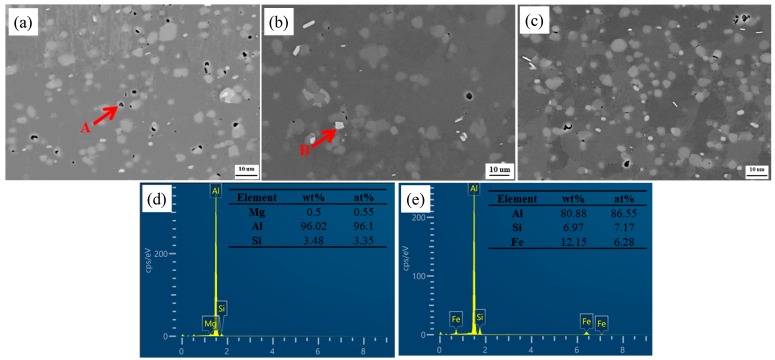
SEM microstructures of base materials (BMs) with different states: (**a**) T1; (**b**) T4; (**c**) T6; (**d**) corresponding energy dispersive spectrometer (EDS) point analysis taken from A second phases in (**a**); (**e**) corresponding EDS point analysis taken from B second phases in (**b**).

The SEM microstructures in the backscatter electron model and the chemical compositions of the main phases of BMs under different states as measured by EDS technique are presented in Figure 2. The main elements of the black particles (A) are composed of Al, Mg, and Si, indicating that these phases are Mg_2_Si phases. The white particles (B) are composed of Al, Fe, and Si, which indicates that these phases are β-Al_5_FeSi phases. As can be seen, the Mg element is mainly presented as Mg_2_Si particles with a size of 1.5 μm under the T1 condition. The β-Al_5_FeSi particles are almost unchanged in all of the BMs. 

**Figure 3:**
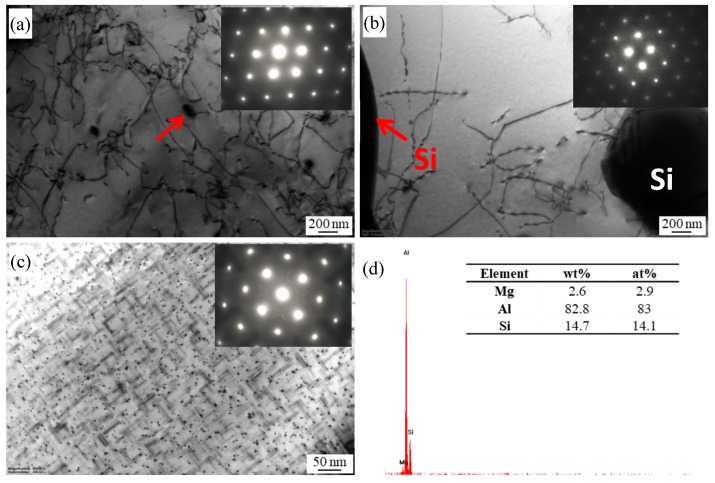
TEM images of BMs with different heat treatment states: (**a**) T1; (**b**) T4; (**c**) T6; (**d**) corresponding EDS point analysis taken from the arrowed second phases in (a).

The representative TEM images of the BM with various states are shown in Figure 3. It can be seen that there are some dislocations and black fine second phases with a size of about 100 nm on the Al matrix under the T1 condition (Figure 3a). According to the EDS analysis (Figure 3d), the black fine second phases (as indicated by the arrow in Figure 3a) contained Mg and Si elements, which can be regarded as Mg_2_Si phases [9,21]. After solution treatment, Figure 3b shows that the Al matrix is free of any precipitate and the electron diffraction pattern demonstrated additional reflexes, which indicates that the Mg_2_Si phases were dissolved into the Al matrix. The microstructures are characterized by fine dot and needle precipitates homogeneously distributing on the Al matrix after solution and artificial aging (Figure 3c). For Al-Mg-Si alloys with high Si content, there are different views on the needle and fine dot precipitates. Some researchers hold the idea that the needle like precipitates are β′′ phases, and the dark dot precipitates are the end of the needle precipitates along the direction of [001] Al [22]. However, Liu et al. believed that the fine dot precipitates are not the cross section of the needle precipitates, and are β′′ [23].

### 3.2. Macrostructure of the Friction Stir Welds

**Figure 4:**
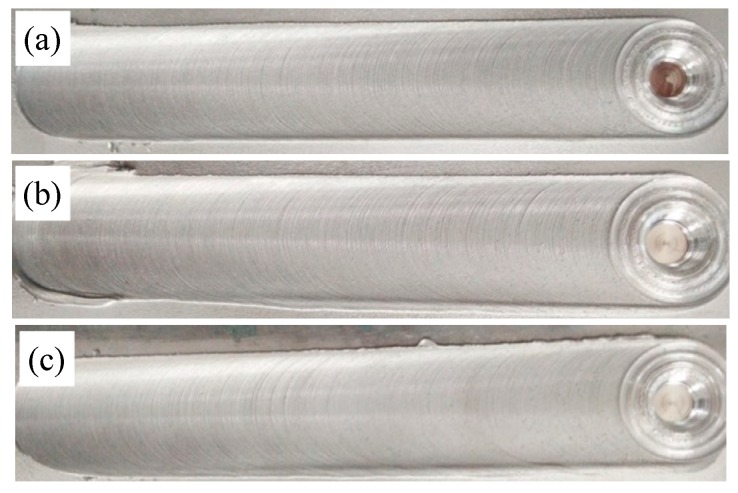
The weld surfaces of the friction stir welds (FSWs) under different states: (**a**) T1; (**b**) T4; (**c**) T6.

The weld surfaces of the friction stir welds (FSWs) in various conditions are shown in Figure 4. The characteristics of the joints are similar to other FSW Al alloys and present as semi-circular features [24]. All joint surfaces are smooth and no welding defects are found, which testifies to the fact that sound and high quality welds can be produced. These results confirm that FSW can be used to prepare Al-Si alloys with sound joints.

**Figure 5:**
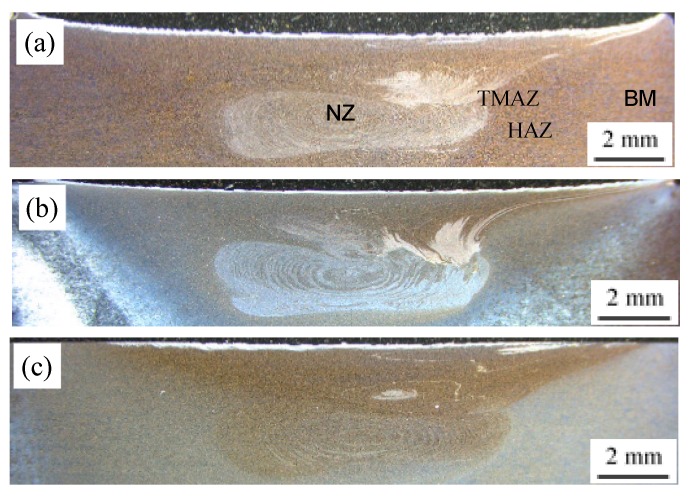
Cross-sectional macrostructures of FSWs under different states: (**a**) T1; (**b**) T4; (**c**) T6 (The advancing side on the right).

Figure 5 shows the macrostructures of FSW joints on the cross-section obtained under T1, T4, and T6 states. It also shows that there are no welding defects in the joints under all conditions. There are four different microstructural zones in the welds, i.e., base material (BM), heat affected zone (HAZ), thermo-mechanically affected zone (TMAZ) and nugget zone (NZ). Similar to most FSW joints in aluminum alloys, all FSW joints produced similar basin-shaped NZs and onion ring structures at the pin-driven area [5]. However, the stir zone of the T6 state is different from that of the T1 and T4 states. The optical contrast is uniformly in the T6 state (Figure 5c), whereas onion ring structures and clear flow patterns can be seen under the T1 and T4 conditions (Figure 5a,b). In the FSW process, this difference may indicate the differences of material flow under T1, T4, and T6 states [24].

### 3.3. Microstructure of the Friction Stir Welds

**Figure 6:**
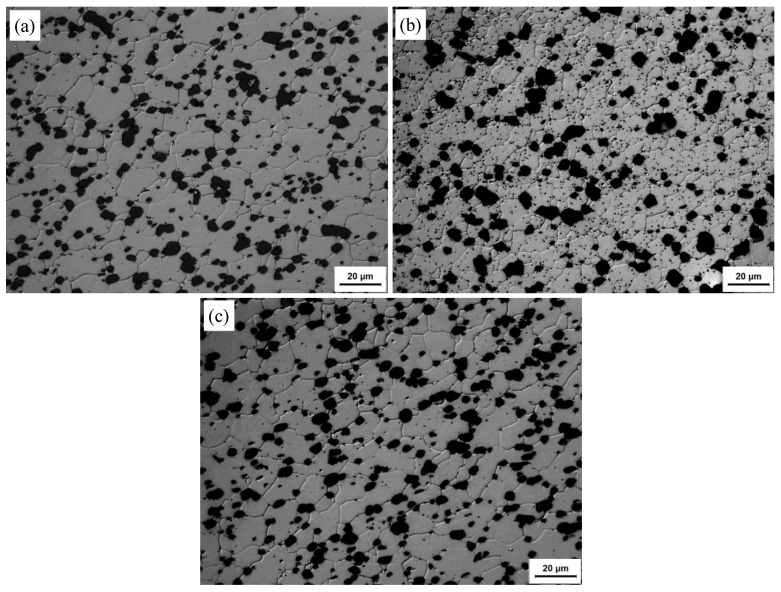
Optical micrographs at the nugget zones (NZs) of FSW joints under different states: (**a**) T1; (**b**) T4; (**c**) T6.

**Figure 7:**
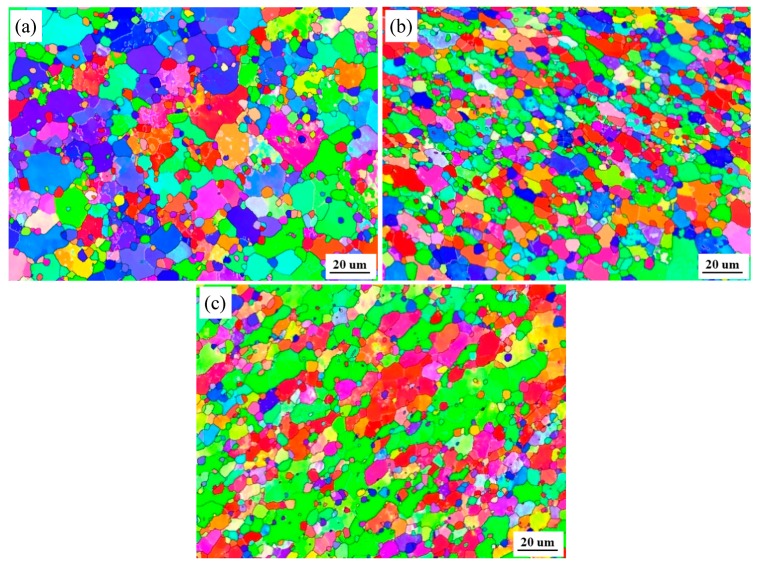
Electron backscattering diffraction (EBSD) maps at the NZs of FSW joints under different states: (**a**) T1; (**b**) T4; (**c**) T6.

The optical microstructures at the NZs of FSW joints in T1, T4, and T6 conditions are shown in Figure 6. In all of the cases studied, the Al matrix microstructures were dominated by equiaxed grains, homogeneously distributed round Si particles. The Si particles are almost unchanged compared with those of the BMs (Figure 1). In the NZs under the T1 state, the Al matrix grain size is ~9.65 μm, which is slightly larger than that of its BM. The Al matrix grain size in the NZs under the T6 state is almost the same as that of its BM or in the NZs under the T1 state. However, the Al matrix grain size is 7.8 μm in the NZs of the T4 state, which is slightly smaller than that in the NZs under the T1 and T6 states. Figure 7 shows the EBSD maps taken from the NZs of FSW joints in the T1, T4, and T6 states. In the images, the low angle boundaries (LABs) (3°~10°) are depicted as white lines, and the high angle boundaries (HABs) (≥10°) are represented by black lines. It can be seen that the HABs dominated in the Al matrix for all of the conditions (Figure 7), and the fraction of the HABs was as high as 75–85%. Generally speaking, the microstructure in the NZs appeared to be completely recrystallized in all of the cases researched. This result was consistent with previous research on friction stir welded aluminum alloys [5,24].

**Figure 8:**
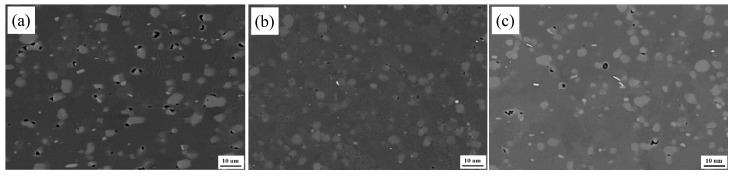
SEM images at the NZs of FSW joints under different states: (**a**) T1; (**b**) T4; (**c**) T6.

**Figure 9:**
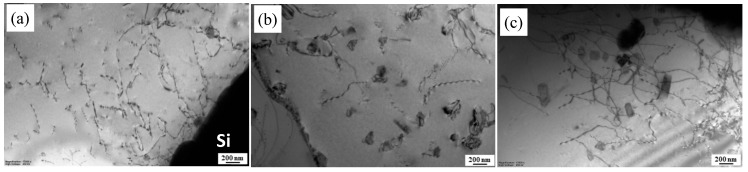
TEM images at the NZs of FSW joints under different initial heat treatment states: (**a**) T1; (**b**) T4; (**c**) T6.

Figure 8 and Figure 9 show the SEM microstructures in the backscatter electron model and TEM images at NZs of FSW joints under T1, T4, and T6 conditions, respectively. Compared with their BM microstructure in Figure 2, the phases of β-Al_5_FeSi are almost unchanged in all conditions. The size of the Mg_2_Si phases is almost unchanged, but their density increased slightly and the small phases disappeared in the NZs under the T1 state, indicating that the small Mg_2_Si phases are aggregation and growth during the FSW process due to the raised temperature (Figure 2a, Figure 3a, Figure 8a, and Figure 9a). From Figure 8b and Figure 9b, it can be seen that there are many fine precipitations from the supersaturated solid solution in the FSW process and homogeneous distribution on the grain boundaries or in the Al matrix grains in the NZs under the T4 condition, which may have hindered the Al matrix grains growth. In the NZs under the T6 condition, the fine dot and needle precipitates in the BM have grown to an average size of 200 nm, or even larger second phases, which is slightly coarser than that in the NZs under the T4 condition (Figure 8c and Figure 9c).

**Figure 10:**
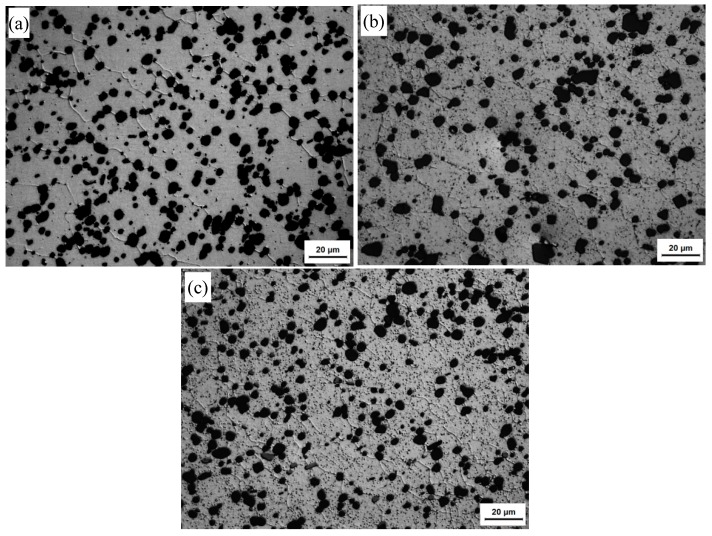
Optical microstructures at the heat affected zones (HAZs) of FSW joints under different states: (**a**) T1; (**b**) T4; (**c**) T6.

**Figure 11:**
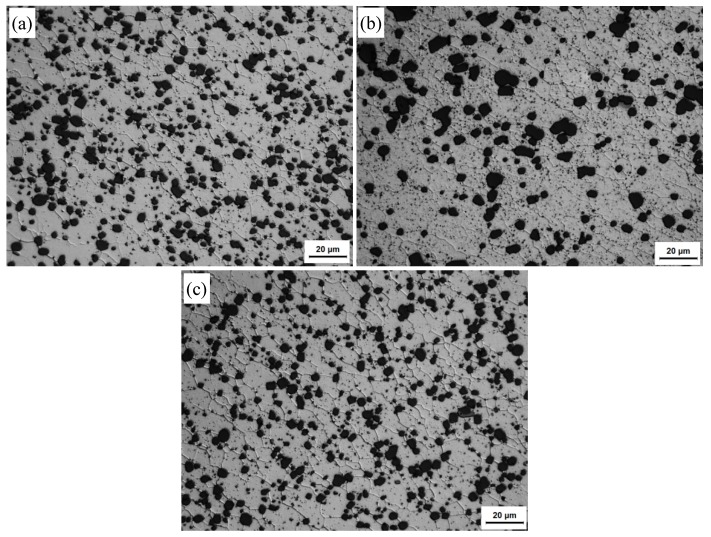
Optical microstructures at the thermo-mechanically affected zones (TMAZs) of FSW joints under different states: (**a**) T1; (**b**) T4; (**c**) T6.

Figure 10 and Figure 11 show, respectively, the optical microstructures of the HAZs and TMAZs of FSW joints under T1, T4, and T6 conditions. The HAZ experienced a thermal cycle. The Al matrix grains are somewhat coarsened in the HAZs under all conditions compared to those in their BMs because of the thermal cycle. The TMAZs have mixed equiaxed and rotated elongated grains, i.e., partial recrystallization morphology in these regions under various conditions (Figure 11), which is consistent with the previous reports [5,25]. The transformation of the second phases in the HAZs and TMAZs is similar to that in the NZs in all conditions, which will not be discussed here.

### 3.4. Hardness Profile

**Figure 12:**
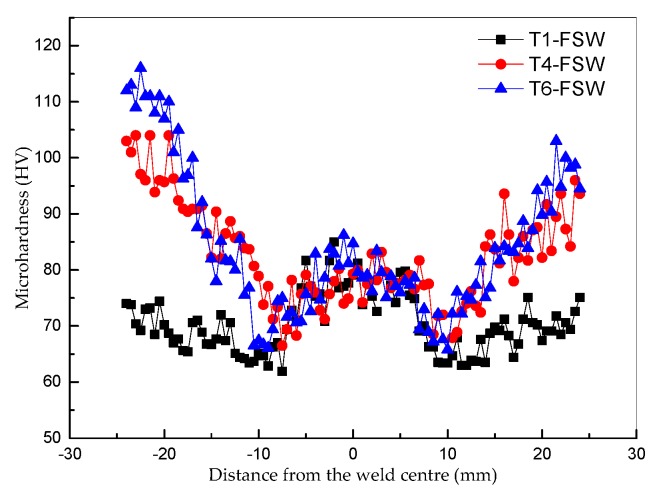
Microhardness profile measurements across the transverse cross-section of the FSW joints under T1, T4, and T6 states.

The mid-thickness microhardness profiles across the transverse cross-section of the FSW joints under various conditions are shown in Figure 12. Obviously, W-shaped hardness profiles were obtained under all conditions. The average microhardness in the NZs, whose width is about 8 mm, equivalent to the diameter of the stirring pin, has almost no variation from the base material and is higher than that in the TMAZs and HAZs under all conditions. The hardness fell through the TMAZs and reached a minimum value of 66 HV at the HAZs in the T4 and T6 states. The minimum hardness is 63 HV at the HAZ in the T1 condition, which is slightly lower than that in the HAZs under the T4 and T6 conditions. The hardness values in the weld joints are significantly lower than those of the BMs under the T4 and T6 states. The FSW process softened the T4 and T6 materials, with the microhardness in the NZs decreasing by nearly 20% and 27% to about 80 HV. However, the hardness values in the NZs are almost equal to or even higher than that of the BM under the T1 state.

It has been reported that the hardness distribution in the weld joints is mainly related to the precipitate distribution in the precipitation hardening aluminum alloys [26]. Since the thermal cycle and plastic deformation in the FSWs, the precipitate particles were coarsened and over-aged in the FSW process [27], which reduced the hardness of the FSW joints to some extent under T1, T4, and T6 states. As can be seen from Figure 6, Figure 8 and Figure 9, the microstructures were almost the same in the NZs of the FSWs except for the small difference in the size of the Al matrix grains and the second phases. Therefore, the average microhardness in the NZs was almost the same under all conditions. The W-shaped hardness distributions could be mainly due to the hardness recovery in the NZs by the post-weld aging because of the peak temperature and duration of the thermal cycle during FSW [28]. This phenomenon has been widely reported in heat-treatable aluminum alloys [6,28,29].

### 3.5. Tensile Properties

Table 1 shows the mechanical properties of the BMs and FSW joints. In the BMs, the strength under the T4 and T6 conditions is much higher than that in the T1 condition. The increase in strength in the T4 and T6 states can be attributed to the Mg_2_Si dissolved in the Al matrix (solution strengthening) and the fine dot and needle precipitates on the Al matrix (precipitation strengthening), respectively (Figure 1 and Figure 2).

It is evident that the ultimate tensile strength (UTS), yield strength (YS), and elongation to fracture of the welds are inferior to those of their BMs. In the T1 condition, the strength of the weld is slightly lower than that of its BM and the joint efficiency is more than 90%. This is because the microstructure in the NZs is almost the same as its BM in the T1 condition. In contrast, the welds at the T4 and T6 states were much softer than their BMs (Table 1). In the T4 and T6 conditions, the strength of the welds and BMs were quite different, and the joint efficiencies for the ultimate tensile strength were only 77.2% and 62%, respectively. This can be attributed to the excess frictional heat dissipation to the welds, resulting in the precipitate phases and coarsening of the Al matrix grains and the strengthening precipitates (Figure 6, Figure 8 and Figure 9), which leads to the elimination of the solid solution strengthening and age hardening effects [26,27]. It can also be seen that the elongations of the FSWs are much lower than those of their BMs in all conditions. This is because the weakest areas of the weld joints are more susceptible to stress and strain concentration under tensile loading [26]. The FSWs under the T1 state exhibited higher elongation of about 13.22%, indicating that the welds have better plasticity than the cast Al-Si alloys [30]. The tensile states are basically consistent with the microhardness results (Figure 12).

In contrast, the weld materials under the T4 and T6 conditions showed almost the same strength regardless of the condition of the BMs (Table 1). In the FSWs under the T1 state, the UTS and YS were slightly lower than those of the T4 and T6 states (Table 1). This result is consistent with the lower microhadness in the HAZ under the T1 state (Figure 12). The phenomenon is related to the similar microstructure, except that there was a slight difference in the size of the second phases, and Al matrix grains formed at the welds under all conditions (Figure 6, Figure 7, Figure 8, Figure 9, Figure 10 and Figure 11).

**Figure 13:**
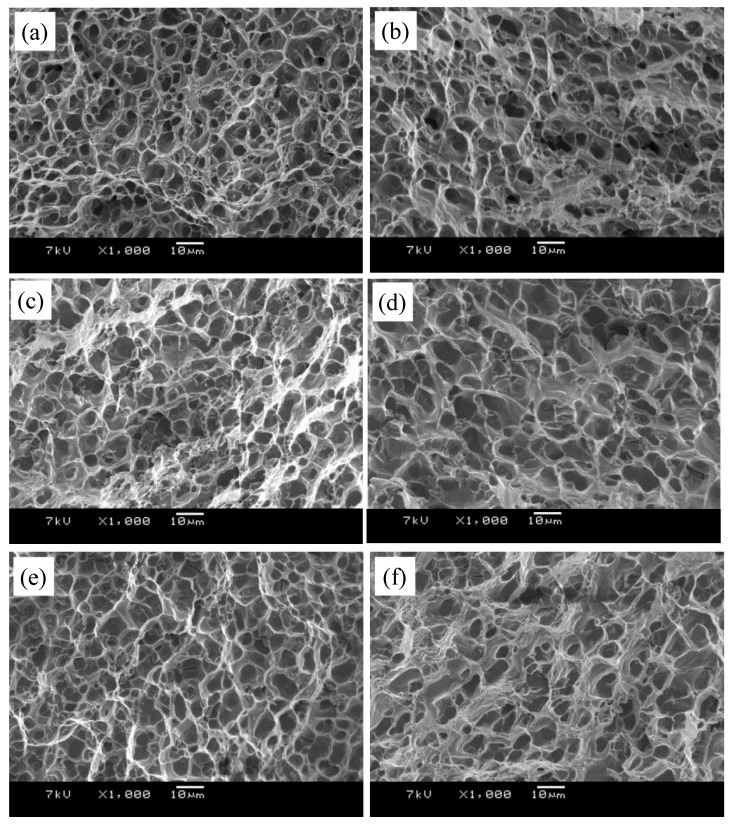
SEM images of the fractography for: (**a**) T1; (**b**) T1-FSW; (**c**) T4; (**d**) T4-FSW; (**e**) T6; (**f**) T6-FSW.

The fracture surfaces of the failed BMs and FSW joints under different conditions are presented in Figure 13. All of the fracture surfaces distributed dimples with different sizes and shapes and tearing edges, indicating that the failures modes are basically ductile fracture. Comparing the fracture morphologies of BMs under different condition, it can be seen that the fracture morphology of the BM under the T1 condition consists of finer, more-uniform, and deeper dimples than that of the T4 and T6 conditions, which are beneficial to the uniform elongation. It can also be seen that the size of the dimples is almost equal to the Si particles. From Figure 1, it is clearly seen that the size of the Si particles was coarsened under the T4 and T6 conditions. Therefore, the BM in the T1 condition exhibited a higher elongation of 21.56% (Table 1).

Comparing the fracture morphology of the FSW joints with that of their BMs, it can be seen that the dimples on the facture surfaces of the FSW joints are more inhomogeneous and shallow (Figure 13), indicating that the elongation of the FSWs are lower. It is also found that the fracture morphology contains more depth and dimples for the FSW joint in the T1 states than in the T4 and T6 conditions. This shows that the ductility of the FSW joints under the T1 state is better than that of the T4 and T6 conditions, which is consist with the results of the elongations in Table 1.

## 4. Conclusions

A wrought Al-11.3Si-0.6Mg alloy was produced by direct-chill (DC) casting and hot extrusion. Heat treatments (Solution treatment (T4) and solution treatment + artificial aging (T6)) before friction stir welded were conducted in order to evaluate the influence of initial heat-treatment states on the microstructures and mechanical properties of the welded joints of the friction stir welded Al-11.3Si-0.6 alloy. Friction stir welding was successfully employed to produce defect-free welds in hot extrusion (T1), T4, and T6 conditions at a welding speed of 100 mm/min and a rotation rate of 800 rpm. The joint efficiency of the ultimate tensile strength under the T1 stats was more than 90%. In the T4 and T6 conditions, the material softened obviously in the welds, leading to the joint efficiency for the ultimate tensile strength being only 77.2% and 62%, respectively. Based on microstructure observations, the high joint efficiency in the T1 state is due to the fact that the microstructure is almost unchanged, except for the slight coarsening of the Al matrix grains and some of the Mg_2_Si phases during the friction stir welding process. On the other hand, substantial weld softening in the T4 state was attributed to the elimination of the solid solution strengthening effect because of the second phases precipitated and growth from the supersaturated solid solution in the friction stir welding process. The weld softening in the T6 state is due to the elimination of the age hardening effect because the fine dot and needle precipitates in the BM have grown to larger second phases.

## Figures and Tables

**Table 1:** Tensile properties of the BMs and the FSW joints.

Sample	Rotation Rate (rpm)	Welding Speed (mm/min)	Yield Strength (MPa)	Ultimate Tensile Strength (MPa)	Elongation (%)	Joint Efficiency (%)
T1	-	-	118.99	197.07	21.56	-
T4	-	-	147.82	246.50	17.70	-
T6	-	-	227.19	304.70	10.30	-
T1-FSW	800	100	102.88	181.41	13.22	92.05
T4-FSW	800	100	111.58	190.35	8.24	77.22
T6-FSW	800	100	105.27	189.01	7.19	62.03
